# Changes in corneal higher-order aberrations during treatment for infectious keratitis

**DOI:** 10.1038/s41598-023-28145-7

**Published:** 2023-01-16

**Authors:** Takehiro Matsumura, Takefumi Yamaguchi, Takanori Suzuki, Yurina Ogiwara, Yoshihiro Takamura, Masaru Inatani, Jun Shimazaki

**Affiliations:** 1 Department of Ophthalmology, Tokyo Dental College Ichikawa General Hospital, 5-11-13 Sugano, Ichikawa, Chiba 272-8513 Japan; 2 Department of Ophthalmology, Faculty of Medical Sciences, University of Fukui, Fukui, Japan; 3 Cornea Center Eye Bank, Tokyo Dental College Ichikawa General Hospital, Ichikawa, Chiba Japan

**Keywords:** Eye diseases, Corneal diseases

## Abstract

This study aimed to quantify the changes in corneal higher-order aberrations (HOAs) before and after treatment for infectious keratitis and verify the correlation between corneal HOAs and visual acuity. Corneal HOAs were analysed using swept-source anterior segment optical coherence tomography (AS-OCT). Ninety-eight eyes of 96 consecutive patients with infectious keratitis treated with topical eye drops were retrospectively evaluated. Corneal HOAs increased with the infection but decreased with infection resolution following antimicrobial treatment. Corneal HOAs became larger as the degree of corneal findings became more severe. The increase in HOAs of the total cornea was correlated with the decrease in visual acuity both before and after treatment (4 mm, ρ = 0.530 and 0.590; 6 mm, ρ = 0.479 and 0.567, respectively; all *P* < 0.0001). Furthermore, pretreatment HOA (anterior, 6 mm), pretreatment logMAR best spectacle-corrected visual acuity, and age were prognostic factors significantly associated with posttreatment visual acuity (β = 0.31, *P* = 0.013; β = 0.36, *P* < 0.0001; and β = 0.35, *P* = 0.0007, respectively) (adjusted R^2^ = 0.474). These results indicate that corneal HOAs quantified using AS-OCT can be used as an objective index to evaluate corneal optical function during the treatment of infectious keratitis.

## Introduction

Infectious keratitis is a major cause of visual impairment and blindness globally^1,2^. Infectious keratitis manifests clinically as corneal epithelial defects and stromal infiltration that can lead to corneal stromal melts and corneal perforation^3^. Patients with severe infectious keratitis due to corneal opacity and irregular astigmatism resulting from excessive degradation of corneal stromal collagen have a poor prognosis. Although irregular astigmatism can be quantitatively assessed by measuring higher-order aberrations (HOAs), it has been difficult to determine aberrations in eyes with strong opacity. The recent development of anterior segment optical coherence tomography (AS-OCT) has enabled the quantitative evaluation of HOA even in such opacified eyes, and the importance of HOA for visual function in post-infectious keratitis scars, as well as in various types of corneal opacities, has been clarified^4–10^. Moreover, this method demonstrated a strong correlation between an increase in corneal HOAs and a decrease in visual acuity^4–10^. However, the changes in HOAs before and after treatment in the clinical course of infectious keratitis from the acute phase to the scar phase remain to be clarified. Furthermore, the effect of HOA alterations on the treatment of infectious keratitis remains elusive.

Thus, this study aimed to investigate the changes in corneal HOAs and topographic map patterns during the clinical course of topical eye drop treatment for infectious keratitis, including bacterial, fungal, *Acanthamoeba*, and herpetic keratitis. We hypothesised that quantitative analysis techniques, such as corneal HOA evaluation using AS-OCT, could be objective diagnostic/prognostic biomarkers for evaluation during infectious keratitis treatment. In addition, we assessed the association between HOA and visual function before and after treatment and determined whether an improvement in visual acuity was correlated with a decrease in HOA after the resolution of infectious keratitis. Finally, clinical factors that determine the visual prognosis of infectious keratitis were also identified.

## Methods

### Study design and patients

This was a retrospective, consecutive case series. Approval was obtained from the Institutional Review Board/Ethics Committee of the Tokyo Dental College Ichikawa General Hospital, Chiba, Japan (approval number: I 15-51). All study procedures adhered to the tenets of the Declaration of Helsinki. The need for written informed consent was waived by the Ethics Review Board of Tokyo Dental College Ichikawa General Hospital owing to the retrospective nature of the study. We disclosed the study protocol to all patients and provided them with the opportunity to refuse participation.

Patients treated for infectious keratitis between January 1, 2019, and December 31, 2020, at the Tokyo Dental College Ichikawa General Hospital were evaluated. Infectious keratitis was diagnosed according to the guidelines for the clinical management of infectious keratitis (2nd edition), which were published by the Japanese Association for Ocular Infection^11^. The diagnosis was determined comprehensively based on patient history; patient background; symptoms; clinical course; slit-lamp microscopy findings; direct microscopic examinations, culture, and immunochromatography of the corneal scrapings; and the response to topical antibiotics, antifungal agents, chlorhexidine, or antiviral agents.

**Figure 1:**
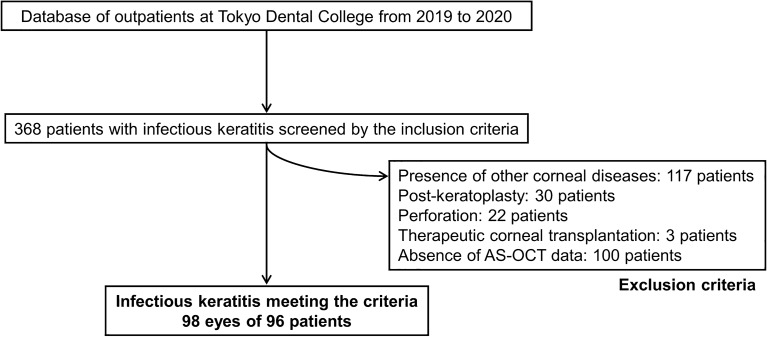
Patient selection flowchart. *AS-OCT* anterior segment optical coherence tomography.

The inclusion criteria were the first episode of infectious keratitis and infectious keratitis treated with topical eye drops, with or without systemic therapy. The exclusion criteria were as follows: (1) eyes with other corneal diseases, such as keratoconus, corneal dystrophies, or bullous keratopathy; (2) presence of other ocular surface diseases, such as conjunctivitis, blepharitis, lacrimal passage disease, Stevens-Johnson syndrome, or mucous membrane pemphigoid; (3) history of corneal surgery, such as corneal transplantation; (4) eyes that progressed to corneal perforation; (5) eyes that required therapeutic corneal transplantation; (6) use of steroid eye drops; (7) absence of AS-OCT data; or (8) refusal to participate in the study (Fig. 1). Diagnosis codes were used to identify the cases that were enrolled in this study. Two experienced corneal specialists (T.M. and T.Y.) reviewed the medical records of all cases to confirm the diagnosis and course of treatment. The following data were collected: age, sex, history of systemic diseases, use of contact lenses, eye drops used for treatment, duration from onset to treatment initiation (days), duration to epithelialization (days) and healing (days), area of corneal infiltration, presence of neovascularization, scar area after healing, and degree of corneal opacity.

### Clinical examination and treatment

Routine examinations, including slit-lamp microscopy, best spectacle-corrected visual acuity (BSCVA), and AS-OCT, were conducted before, during treatment, and after the resolution of the infection. In the treatment of infectious keratitis, the decision to examine the AS-OCT images at every visit was at the discretion of the treating ophthalmologists.

Corneal scrapings for culture and smears were performed at the first visit to identify the causative microorganism. The foci of the corneal infection were scraped, and the scrapings were directly inoculated into blood, Sabouraud dextrose, and GAM agar, followed by incubation for 3 weeks. In addition, corneal scrapings collected similarly were used for smears for direct microscopic examinations. Culture for *Acanthamoeba* and immunochromatography for herpes were performed additionally only when suspected clinically. Infectious keratitis was treated according to the guidelines for the clinical management of infectious keratitis^11^. Empirical treatment was initiated immediately based on the initial assessment without waiting for culture results. Patients were then instructed to revisit within a few days to assess the response to the antimicrobial agents and determine whether the diagnosis and current treatment were appropriate. Initial treatment was continued if effective. The culture and drug susceptibility results, if available, were used to determine whether the drug should be changed. If the initial treatment was found to be ineffective, the diagnosis and treatment strategy were assessed again. Topical antimicrobial treatment was administered until the resolution of infectious keratitis. Healing was defined as the resolution of inflammation and the scarring of the corneal lesions, with no recurrence of the infection after finishing treatment. In the treatment against fungal or Acanthamoeba infection, antibiotic eye drops were used to control mixed infections in addition to the drugs used to treat the causative microorganism. Antibiotic eye drops were used up to 2–3 times per day for this purpose. Therapeutic agents against the causative microorganism were administered with intensive treatment every 1–2 h, and the patient’s condition was assessed to determine whether there was any improvement.

The degree of corneal opacity was graded on the basis of slit-lamp examination with previously described systems^4,12,13^ as follows: grade 0, clear or trace haze; grade 1, mild opacity; grade 2, moderately dense opacity partially obscuring the details of the iris; and grade 3, severely dense opacity obscuring the details of the intraocular structures. BSCVAs were recorded as decimal acuities and converted to logarithm of the minimum angle of resolution (logMAR) units for statistical analyses. BSCVA and corneal HOA were assessed at three time points: at the first visit to our hospital, 2 weeks after initiating topical antimicrobial agents, and 1 month after the resolution of infectious keratitis.

### Anterior segment optical coherence tomography

The eyes of patients with infectious keratitis were examined using AS-OCT (SS-1000 or SS-2000; CASIA or CASIA2, Tomey, Nagoya, Japan). This swept-source OCT uses a wavelength of 1310 nm and provides axial resolution of 10 μm, transverse resolution of 30 μm, and a scan velocity of 30,000 or 50,000 A-scans per second. Sixteen rotating AS-OCT scans were used to reconstruct three-dimensional models of the entire corneal structure. The CASIA system corrects distortions in the AS-OCT images based on the refractive index of the anterior surface. All participants were examined until at least two sets of sufficient images were obtained. Two corneal specialists (T.M. and T.Y.) carefully assessed all AS-OCT images to ensure that the surface digitalization recognised by the automated built-in software was correct. Zernike coefficients were calculated using Zernike analysis, as previously reported^14^. In brief, the anterior and posterior corneal surfaces were reconstructed using the corneal height data as a three-dimensional model. The anterior, posterior, and total corneal aberrations at diameters of 4 and 6 mm were calculated separately using the installed ray-tracing software. The refractive indices of the cornea and aqueous humor were set at 1.376 and 1.336, respectively. Wavefront aberration was expanded with normalised Zernike polynomials up to the eighth order. HOA was defined as the root mean square (RMS) of the third- to eighth-order Zernike coefficients. Spherical aberration (SA) was defined as the RMS of ${\text{Z}}_{4}^{0}$ (SA) and ${\text{Z}}_{6}^{0}$ (secondary SA). Coma aberration (coma) was calculated as the RMS of ${\text{Z}}_{3}^{-1}$ and ${\text{Z}}_{3}^{1},$ using the same method as in previous studies^4–6^.

Topographic map patterns were defined as follows, and alterations were assessed before and after treatment^4–6^. The asymmetric pattern was a mirror-image with blue-red asymmetric colour in the topographical map of the anterior surface: one half with a keratometric value of > 50 dioptres and the other half with a keratometric value of < 35 dioptres. The protrusion pattern was a centrally steep zone with a keratometric value of > 50 dioptres on the anterior surface. The keratoconus-like pattern was defined as inferior-superior or nasal-temporal dioptric asymmetry, with corneal thinning in the steepening area and without a flat area on the opposite side, as in keratoconus. The flattening pattern involved a flattened cornea with a keratometric value of < 40 dioptres on the anterior corneal surface. Eyes with minimal alterations on the anterior surface and an irregular posterior surface were defined as having a posterior irregular pattern. The minimal change pattern had an almost normal topographic map with minimal irregular astigmatism. Corneal thinning or thickening was defined as corneal thickness on a pachymetry map at lesions < 350 μm or > 700 μm, respectively.

### Statistical analyses

The Wilcoxon signed-rank test was used to compare the differences in corneal HOAs or logMAR BSCVAs before and after treatment. The duration from infection onset to treatment initiation for each aetiology, HOAs among the different corneal opacity grades or aetiologies, or logMAR BSCVAs among the different topographic map patterns were compared using the Steel–Dwass test for non-parametric multiple comparison. The differences in corneal HOAs with or without central corneal lesions were compared using the Mann–Whitney U test. Spearman correlation analysis was used to evaluate the possible correlations between logMAR BSCVA and HOAs, corneal opacity area and HOAs, or ΔlogMAR BSCVA and ΔHOA ratio. One patient each with proliferative diabetic retinopathy, advanced glaucoma, and moderate-to-severe cataract were excluded from the analysis of visual acuity. Furthermore, pretreatment clinical prognostic factors associated with posttreatment visual acuity were determined with multivariable analysis using multiple regression analysis models. Baseline characteristics, including age, sex, patient background factors, aetiology of infectious keratitis, duration from onset to treatment initiation, duration to epithelialization, duration to healing, the extent of corneal infiltration, location of the corneal infiltration, the presence or absence of neovascularization, corneal opacity grade, pretreatment BSCVA, corneal HOAs, and topographic map pattern, were evaluated as possible predictors associated with posttreatment visual acuity. Values are presented as the median (interquartile range, IQR). All statistical analyses were performed using JMP 10 software (SAS Institute Inc., Tokyo, Japan). *P* values < 0.05 were considered statistically significant.

## Results

### Patient characteristics

**Figure 2:**
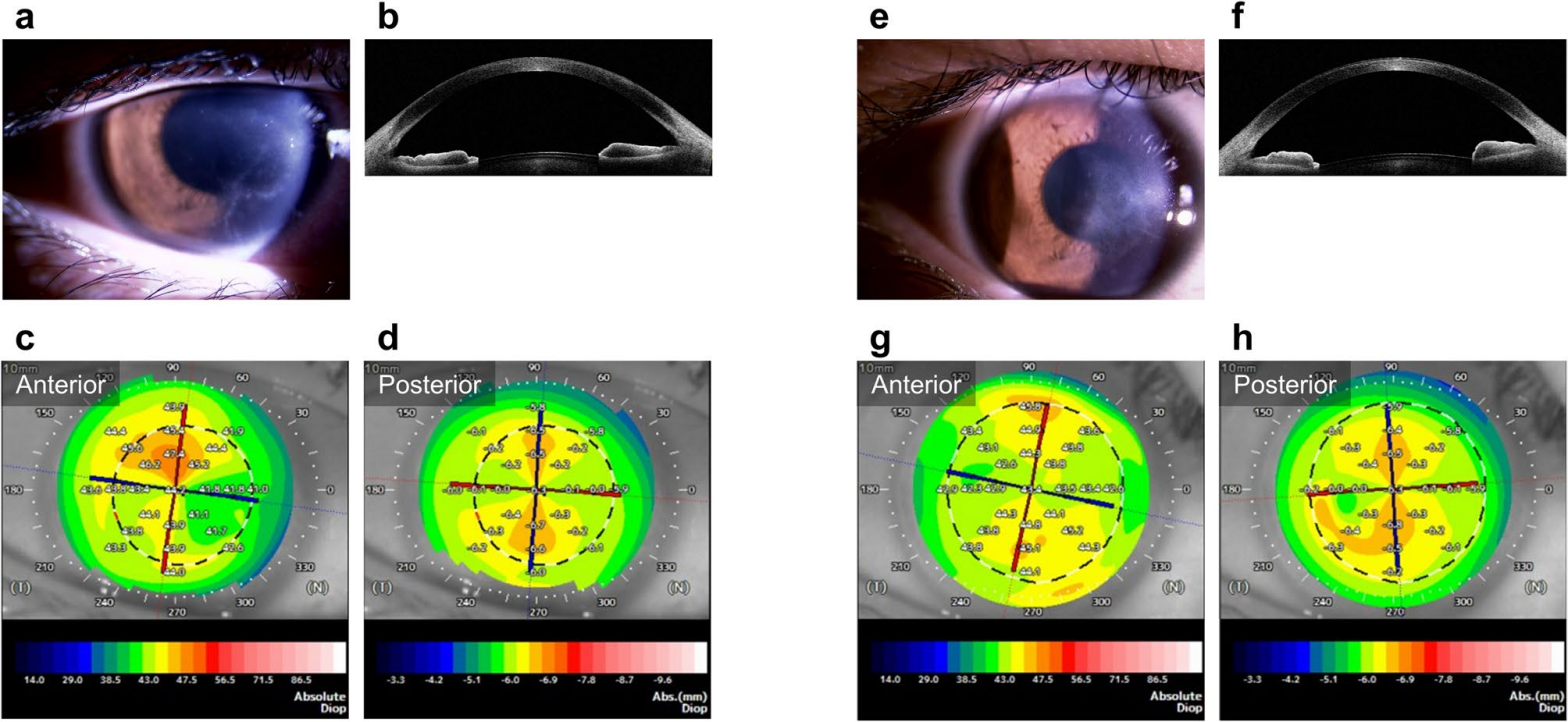
Slit-lamp and anterior segment optical coherence tomography images of a representative case of *Acanthamoeba* keratitis. (**a,b**) Before treatment, radial keratoneuritis is observed near the centre of the cornea to the nasal and inferior corneas. (**c,d**) The corneal higher-order aberration (HOA) (total, 6 mm) is 1.55 μm, and the logMAR best spectacle-corrected visual acuity (BSCVA) is 0.046. (**e–h**) The patient is treated with ophthalmic drops, and healed with corneal scarring. The corneal HOA (total, 6 mm) is 0.98 μm, and the logMAR BSCVA improved to − 0.08 after treatment, although a mild scar remains near the centre of the cornea.

**Figure 3:**
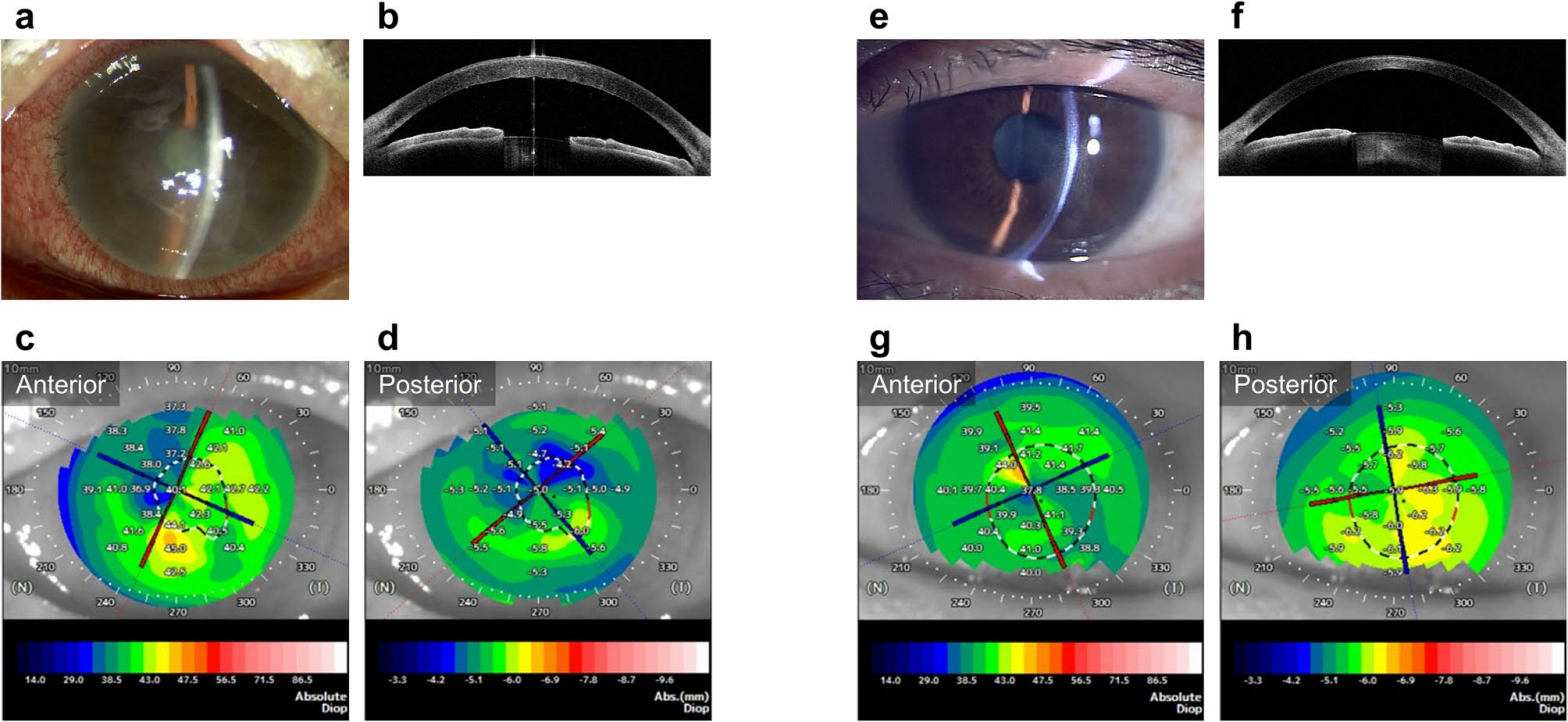
Slit-lamp and anterior segment optical coherence tomography images of a representative case of bacterial keratitis. (**a,b**) Before treatment, corneal infiltration and edema are observed near the centre of the cornea. (**c,d**) The corneal higher-order aberration (HOA) (total, 6 mm) is 3.20 μm, and the logMAR best spectacle-corrected visual acuity (BSCVA) is 1.80. (**e–h**) The patient is treated with antibiotic eye drops and healed with a mild scar near the centre of the cornea. However, the corneal HOA (total, 6 mm) after treatment is 2.41 μm, and the logMAR BSCVA is only 0.40 due to irregular astigmatism.

In total, 98 eyes of 96 patients were evaluated. Table 1 summarizes the patient characteristics. The median patient age was 50 (34–65) years; 46 patients were females and 52 were males. Overall, 47 patients used contact lenses, and six, four, and three patients had diabetes mellitus, after trauma, and atopy, respectively. The aetiologies of infectious keratitis were bacteria in 62 patients, fungi in 14, *Acanthamoeba* in 14, and herpes simplex virus in eight. The median duration from infection onset to treatment initiation was 3 (1–14) days. After starting treatment, the median duration to epithelialization was 20 (9–36) days, and the median duration to healing was 50 (28–90) days. The median number of drugs used for treatment was 3 (2–4). The following drugs were used in the treatment: levofloxacin, cefmenoxime, dibekacin, chloramphenicol and colistin, and ofloxacin ointment as antibiotics; voriconazole, natamycin, and micafungin as antifungal agents; acyclovir ointment as an antiviral agent; and chlorhexidine as a disinfectant. There were three cases in which the treatment was adjusted according to the culture results. *Pseudomonas aeruginosa* were detected in the cultures of all cases, and the antibiotic eye drops were adjusted according to the clinical course and the drug susceptibility results. Systemic administration was combined with the use of topical eye drops in 11 cases. The systemic antimicrobial agents used were the antifungal agents itraconazole or fluconazole, or the antiviral agent valacyclovir. Figures 2 and 3 show the representative cases of infectious keratitis.

**Table 1:** Patient characteristics.

**Characteristics**	
Number of eyes, n	98
Age (years)	50 (34–65)
Sex (female/male), n	46/52
Patients background factor, n	
Use of contact lens	47
Diabetes mellitus	6
Post-trauma	4
Atopy	3
Aetiology of infectious keratitis, n	
Bacteria	62
Fungi	14
* Acanthamoeba*	14
Herpes simplex virus	8
Duration from onset to treatment initiation (days)	3 (1–14)
Duration to epithelialization (days)	20 (9–36)
Duration to healing (days)	50 (28–90)
Number of medications	3 (2–4)
Pretreatment corneal findings	
Area of infiltration (mm^2^),	10.2 (4.7–25.3)
Location of the corneal lesion, n	
Central/paracentral/peripheral	55/85/34
Neovascularization before treatment (+ / −)	12/86
Corneal opacity grade before treatment, n Grade 1/Grade 2/Grade 3	8/23/67
Posttreatment corneal findings	
Scar area after healing (mm^2^)	3.0 (0.8–12.0)
Neovascularization after treatment (+ / −)	16/82
Corneal opacity grade after treatment, n Grade 0/Grade 1/Grade 2/Grade 3	16/42/27/13
Corneal thickening after treatment, n	4
Corneal thinning after treatment, n	24
BSCVA before treatment (logMAR units)	1.11 (0.30–1.76)
BSCVA after treatment (logMAR units)	0.10 (− 0.07–0.52)

The values are presented as median (interquartile range).

The location of the corneal lesion was defined as 3 mm from centre of the cornea for central, within 3–6 mm for paracentral, and beyond 6 mm for peripheral.

*BSCVA* best spectacle-corrected visual acuity, *logMAR* logarithm of the minimum angle of resolution.

Corneal findings before treatment included a median area of corneal infiltration of 10.2 (4.7–25.3) mm^2^, with central, paracentral, and peripheral corneal involvement in 55, 85, and 34 eyes, respectively (there was overlapping of multiple areas). The pretreatment corneal opacity grade was grade 1, grade 2, and grade 3 in eight, 23, and 67 eyes, respectively. Neovascularization was present in 12 eyes. The duration from infection onset to the initiation of correct treatment was significantly longer in patients with fungal keratitis (FK) and *Acanthamoeba* keratitis (AK) than that in patients with bacterial keratitis (BK) (FK vs. BK: 15.5 [2.5–48.75] days vs. 2 [1–5] days, *P* = 0.0051; AK vs. BK: 23.5 [13.25–40] days vs. 2 [1–5] days, *P* < 0.0001). No statistically significant correlations were observed between the duration from the onset to treatment initiation and the area of corneal infiltration or corneal opacity grade for any aetiology.

After healing, the median area of corneal scarring was 3.0 (0.8–12.0) mm^2^, and the corneal opacity grades were 0, 1, 2, and 3 in 16, 42, 27, and 13 eyes, respectively; neovascularization was present in 16 eyes. After scar healing, corneal thickening and corneal thinning were observed in four and 24 eyes, respectively. Visual acuity significantly improved from 1.11 (0.30–1.76) logMAR before treatment to 0.10 (− 0.07–0.52) logMAR after treatment (*P* < 0.0001).

### Longitudinal changes in corneal higher-order aberrations during treatment

Table 2 shows the longitudinal changes in the HOAs, SA, and coma of Zernike terms at 4- and 6-mm diameters during the treatment of infectious keratitis. All HOA parameters, including HOAs, SA, and coma (total/anterior/posterior, 4 mm/6 mm), were significantly decreased after treatment (all *P* < 0.0001).

**Table 2:** Longitudinal changes in corneal higher-order aberrations during treatment.

	Before treatment	During treatment	After treatment	*P* value
HOA (4 mm)				
Total	1.97 (1.17–3.88)	2.00 (1.05–3.85)	1.15 (0.48–2.07)	< 0.0001
Anterior	1.87 (0.95–3.73)	2.04 (0.87–3.90)	1.17 (0.48–2.17)	< 0.0001
Posterior	0.61 (0.39–0.87)	0.47 (0.23–0.89)	0.17 (0.09–0.41)	< 0.0001
HOA (6 mm)				
Total	3.17 (2.09–7.31)	3.74 (1.97–7.28)	2.11 (0.97–4.48)	< 0.0001
Anterior	3.30 (1.83–7.64)	3.84 (1.99–7.29)	2.30 (0.98–4.56)	< 0.0001
Posterior	0.90 (0.61–1.49)	0.79 (0.44–1.27)	0.29 (0.19–0.64)	< 0.0001
SA (4 mm)				
Total	1.14 (0.69–2.46)	1.26 (0.52–2.33)	0.61 (0.30–1.17)	< 0.0001
Anterior	1.08 (0.61–2.36)	1.18 (0.54–2.32)	0.59 (0.32–1.25)	< 0.0001
Posterior	0.34 (0.18–0.46)	0.23 (0.10–0.43)	0.08 (0.05–0.19)	< 0.0001
SA (6 mm)				
Total	1.79 (0.95–3.87)	2.09 (1.02–3.54)	1.27 (0.53–2.54)	< 0.0001
Anterior	1.66 (0.90–3.72)	2.26 (0.96–3.87)	1.24 (0.60–2.51)	< 0.0001
Posterior	0.50 (0.30–0.78)	0.39 (0.22–0.62)	0.16 (0.13–0.29)	< 0.0001
Coma (4 mm)				
Total	1.43 (0.82–2.93)	1.58 (0.78–3.13)	0.88 (0.37–1.73)	< 0.0001
Anterior	1.42 (0.70–2.88)	1.53 (0.68–3.18)	0.81 (0.34–1.68)	< 0.0001
Posterior	0.49 (0.29–0.75)	0.38 (0.21–0.73)	0.14 (0.07–0.33)	< 0.0001
Coma (6 mm)				
Total	2.54 (1.43–5.68)	2.88 (1.62–6.21)	1.72 (0.78–3.62)	< 0.0001
Anterior	2.53 (1.47–5.74)	2.79 (1.52–6.44)	1.72 (0.80–3.75)	< 0.0001
Posterior	0.73 (0.52–1.24)	0.65 (0.33–1.08)	0.24 (0.14–0.50)	< 0.0001

The values are presented as median (interquartile range) (μm).

*P* values were compared between values before and after treatment using the Wilcoxon signed-rank test.

*Coma* coma aberration, *SA* spherical aberration, *HOA* higher-order aberration.

Table 3 shows the corneal HOAs of Zernike terms of 4- and 6-mm diameters for each of the aetiologies. With respect to the HOA changes by aetiology, all pretreatment HOAs, including total, anterior, and posterior, were large in FK and herpes simplex keratitis (HSK) at 4-mm/6-mm diameters. However, no significant differences were observed between the other aetiologies. Moreover, all pretreatment HOAs, including total, anterior, and posterior, were the smallest in AK at 4-mm/6-mm diameters. However, no significant differences were observed between the other aetiologies. Posttreatment total and anterior corneal HOAs at 4-mm/6-mm diameters were significantly larger in FK than those in AK (4 mm: total, *P* = 0.027; anterior, *P* = 0.011; 6 mm: total, *P* = 0.031; anterior, *P* = 0.031). Posttreatment posterior corneal HOAs at 4-mm/6-mm diameters were significantly larger in FK than in BK (4 mm, *P* = 0.039; 6 mm, *P* = 0.048).

**Table 3:** Corneal higher-order aberrations before and after treatment for each of the aetiologies.

	BK (n = 62)	FK (n = 14)	AK (n = 14)	HSK (n = 8)	*P* value					
					BK vs. FK	BK vs. AK	BK vs. HSK	FK vs. AK	FK vs. HSK	AK vs. HSK
Before treatment										
HOA (4 mm)										
Total	1.70 (0.91–3.72)	2.79 (1.61–5.76)	1.42 (1.13–2.72)	4.60 (2.27–6.21)	0.37	0.95	0.19	0.34	0.89	0.14
Anterior	1.62 (0.74–3.36)	2.67 (1.33–6.09)	1.31 (1.12–2.90)	4.40 (2.39–6.51)	0.25	0.99	0.095	0.36	0.86	0.18
Posterior	0.59 (0.35–0.85)	0.63 (0.45–1.45)	0.54 (0.23–0.83)	0.91 (0.59–1.61)	0.89	0.96	0.29	0.83	0.79	0.18
HOA (6 mm)										
Total	2.93 (1.84–5.97)	5.66 (2.66–16.2)	2.56 (1.85–3.75)	8.61 (5.10–9.63)	0.24	0.92	0.074	0.17	0.97	0.024
Anterior	2.70 (1.73–6.00)	5.66 (2.24–16.8)	2.56 (1.74–4.03)	8.80 (5.13–10.7)	0.15	0.98	0.076	0.16	0.99	0.061
Posterior	0.89 (0.50–1.52)	0.89 (0.71–2.85)	0.86 (0.48–1.04)	1.22 (0.91–2.04)	0.88	0.77	0.50	0.70	0.94	0.16
After treatment										
HOA (4 mm)										
Total	0.98 (0.45–1.77)	1.95 (1.14–2.92)	0.70 (0.29–1.25)	1.51 (0.57–3.10)	0.053	0.57	0.76	0.027	0.99	0.29
Anterior	0.94 (0.46–1.81)	1.76 (1.16–2.78)	0.73 (0.29–1.18)	1.51 (0.65–3.63)	0.066	0.53	0.68	0.011	1.00	0.22
Posterior	0.15 (0.07–0.30)	0.36 (0.15–0.71)	0.16 (0.07–0.42)	0.55 (0.20–1.12)	0.039	0.99	0.038	0.26	0.77	0.14
HOA (6 mm)										
Total	2.04 (0.94–3.57)	3.50 (2.26–5.59)	1.95 (0.69–2.87)	4.31 (1.16–6.15)	0.061	0.66	0.50	0.031	0.99	0.29
Anterior	2.07 (0.95–3.88)	3.60 (2.25–5.80)	1.77 (0.78–3.01)	4.56 (1.28–5.98)	0.066	0.71	0.46	0.031	1.00	0.29
Posterior	0.24 (0.17–0.43)	0.51 (0.26–1.41)	0.30 (0.18–0.71)	0.72 (0.30–1.28)	0.048	0.93	0.072	0.39	0.99	0.32

The values are presented as median (interquartile range) (μm).

*P* values were compared among each aetiology using the Steel–Dwass test for non-parametric multiple comparison.

*AK* *Acanthamoeba* keratitis, *BK* bacterial keratitis, *FK* fungal keratitis, *HOA* higher-order aberration, *HSK* herpes simplex keratitis.

### Corneal higher-order aberrations based on the corneal findings

Supplementary Table S1 shows the value of HOAs stratified by the corneal opacity grade for infectious keratitis. Before treatment, there were significant differences in all corneal HOAs (total/anterior/posterior, 4 mm/6 mm) between opacity grades 1 and 3 (all *P* < 0.05). After treatment, there were significant differences in all corneal HOAs (total/anterior/posterior, 4 mm/6 mm) between opacity grades 0 and 3 (all *P* < 0.01). There were also significant differences in the total and anterior corneal HOAs at 4-mm/6-mm diameters between opacity grades 0 and 1 and between opacity grades 0 and 2 (all *P* < 0.05). Furthermore, there were significant differences in the total and posterior corneal HOAs at 4-mm/6-mm diameters between opacity grades 1 and 3 (all *P* < 0.05). Supplementary Table S2 shows the correlation between the corneal HOAs and corneal opacity area before and after treatment. There was a significant positive correlation between the infiltration or scar area and all HOAs, including total, anterior, and posterior HOAs at 4- and 6-mm diameters (all *P* < 0.0001). Supplementary Table S3 shows the HOAs based on the presence or absence of central corneal infiltration or scarring in patients with infectious keratitis. All HOAs (total/anterior/posterior, 4 mm/6 mm) were significantly larger in patients with central corneal infiltration or scarring than those in patients without central corneal infiltration or scarring (all *P* < 0.01).

### Corneal topographic map patterns before and after treatment

In the total eyes, the most common alteration in the topographic maps before treatment was the asymmetric pattern (26 eyes, 27%), followed by the posterior irregular (25 eyes, 26%) and protrusion (21 eyes, 21%) patterns (Table 4). Meanwhile, the most common alteration after treatment was the minimal change pattern (50 eyes, 51%), followed by the asymmetric (24 eyes, 25%) and protrusion (10 eyes, 10%) patterns. The posterior irregular pattern was less frequent (three eyes, 3%). LogMAR visual acuity was significantly worse in eyes with asymmetric and protrusion patterns than in eyes with a minimal change pattern, both before (*P* = 0.011 and *P* = 0.030, respectively) and after (*P* = 0.0002 and *P* = 0.0013, respectively) treatment.

**Table 4:** Corneal topographic map patterns before and after treatment.

	Total, n (%)	BK	FK	AK	HSK
Before treatment					
Asymmetric pattern	26 (27)	14 (23)	8 (57)	2 (14)	2 (25)
Protrusion pattern	21 (21)	12 (19)	1 (7)	3 (21)	5 (63)
Keratoconus-like pattern	7 (7)	4 (6)	2 (14)	1 (7)	0
Flattening pattern	1 (1)	1 (2)	0	0	0
Posterior irregular pattern	25 (26)	17 (27)	3 (22)	4 (29)	1 (12)
Minimal change pattern	18 (18)	14 (23)	0	4 (29)	0
After treatment					
Asymmetric pattern	24 (25)	11 (18)	5 (36)	4 (29)	4 (50)
Protrusion pattern	10 (10)	5 (8)	3 (21)	1 (7)	1 (12.5)
Keratoconus-like pattern	8 (8)	6 (10)	2 (14)	0	0
Flattening pattern	3 (3)	1 (1)	0	1 (7)	1 (12.5)
Posterior irregular pattern	3 (3)	2 (3)	0	1 (7)	0
Minimal change pattern	50 (51)	37 (60)	4 (29)	7 (50)	2 (25)

*AK* *Acanthamoeba* keratitis, *BK* bacterial keratitis, *FK* fungal keratitis, *HSK* herpes simplex keratitis.

### Correlations between corneal higher-order aberrations and visual acuities before and after treatment

Table 5 shows the correlation between HOAs and visual acuity before and after treatment. Before treatment, there was a significant positive correlation between the logMAR visual acuities and all HOAs, including total, anterior, and posterior HOAs at 4- and 6-mm diameters (all *P* < 0.001). For pretreatment SA and coma, there was also a significant positive correlation between logMAR visual acuities and all SA or coma at 4- and 6-mm diameters (all *P* < 0.01).

**Table 5:** Correlations between corneal higher-order aberrations and visual acuities before and after treatment.

	Before treatment		After treatment	
	ρ	*P* value	ρ	*P* value
HOA (4 mm)				
Total	0.530	< 0.0001	0.590	< 0.0001
Anterior	0.532	< 0.0001	0.600	< 0.0001
Posterior	0.422	< 0.0001	0.647	< 0.0001
HOA (6 mm)				
Total	0.479	< 0.0001	0.567	< 0.0001
Anterior	0.479	< 0.0001	0.570	< 0.0001
Posterior	0.374	0.0002	0.646	< 0.0001
SA (4 mm)				
Total	0.543	< 0.0001	0.515	< 0.0001
Anterior	0.518	< 0.0001	0.511	< 0.0001
Posterior	0.380	0.0001	0.674	< 0.0001
SA (6 mm)				
Total	0.440	< 0.0001	0.504	< 0.0001
Anterior	0.417	< 0.0001	0.491	< 0.0001
Posterior	0.328	0.0011	0.514	< 0.0001
Coma (4 mm)				
Total	0.501	< 0.0001	0.632	< 0.0001
Anterior	0.521	< 0.0001	0.635	< 0.0001
Posterior	0.435	< 0.0001	0.643	< 0.0001
Coma (6 mm)				
Total	0.467	< 0.0001	0.593	< 0.0001
Anterior	0.493	< 0.0001	0.590	< 0.0001
Posterior	0.384	< 0.0001	0.667	< 0.0001

One patient each with proliferative diabetic retinopathy, advanced glaucoma, and moderate-to-severe cataract were excluded from the analysis of visual acuity.

*Coma* coma aberration, *SA* spherical aberration, *HOA* higher-order aberration, ρ Spearman’s rank correlation coefficient.

After treatment, there was a significant positive correlation between the logMAR visual acuities and all HOA parameters, including HOAs, SA, and coma at 4- and 6-mm diameters (all *P* < 0.0001).

### Correlations between corneal higher-order aberration change and visual improvement after treatment

Analysis of the correlation between the HOA changes in the cornea and visual improvement before and after treatment showed that there was a significant positive correlation between ΔHOA ratio and ΔlogMAR BSCVA in all HOAs, including total, anterior, and posterior HOAs at the 6-mm diameter (total, ρ = 0.228, *P* = 0.029; anterior, ρ = 0.217, *P* = 0.038; and posterior, ρ = 0.252, *P* = 0.015; Supplementary Table S4).

### Pretreatment clinical factors associated with posttreatment visual acuity

Multiple regression analysis of the pretreatment clinical factors for visual prognosis demonstrated that posttreatment logMAR BSCVA was significantly associated with pretreatment HOA (anterior, 6 mm) (B = 0.020 [0.0043–0.036], β = 0.31, *P* = 0.013), pretreatment logMAR BSCVA (B = 0.34 [0.21–0.48], β = 0.36, *P* < 0.0001), and age (B = 0.0083 [0.0036–0.013], β = 0.35, *P* = 0.0007) (adjusted R^2^ = 0.474, Table 6).

**Table 6:** Multivariable analysis to identify pretreatment clinical factors associated with posttreatment visual acuity.

	B (95% CI)	β	*P* value
Pretreatment HOA (anterior, 6 mm)	0.020 (0.0043–0.036)	0.31	0.013
Pretreatment logMAR BSCVA	0.34 (0.21–0.48)	0.36	< 0.0001
Age	0.0083 (0.0036–0.013)	0.35	0.0007
Adjusted R^2^	0.474		

One patient each with proliferative diabetic retinopathy, advanced glaucoma, and moderate-to-severe cataract were excluded from the analysis of visual acuity.

*BSCVA* best spectacle-corrected visual acuity, *HOA* higher-order aberration, *logMAR* logarithm of the minimum angle of resolution, *B* partial regression coefficient, *CI* confidence interval, β  standardised partial regression coefficient, *R*^*2*^ coefficient of determination.

## Discussion

The changes in HOA during the clinical course of infectious keratitis, from the acute phase to the scar phase, remain to be clarified. In this study, corneal HOAs increased with the infection and then decreased significantly after treatment. Corneal HOAs became larger as the degree of corneal opacity due to infectious keratitis, infiltration in the acute phase and scarring in the resolution phase, became more severe. Furthermore, corneal HOAs were positively correlated with the extent of corneal opacity and the presence of central corneal lesions. With respect to visual function, corneal HOAs showed a significant correlation with logMAR visual acuity, both before and after treatment for infectious keratitis. Moreover, in corneal HOA at the 6-mm diameter, there was a correlation between the improvement in HOA and improvement in visual acuity.

Patients with post-infectious keratitis have larger total, anterior, and posterior corneal HOAs than normal controls, suggesting that post-infectious scars not only scatter but also induce morphological changes in the anterior and posterior corneal surfaces, increasing corneal HOAs^4^. In the current study, larger corneal HOAs were associated with poorer visual acuity; conversely, smaller corneal HOAs were associated with better visual acuity, which is consistent with a previous report^4^. Furthermore, corneal HOA was found to be correlated with logMAR visual acuity, even in the pretreatment state. These findings suggest that in addition to visual acuity, HOA, as an objective marker of irregular corneal astigmatism, may be a useful index in the evaluation of visual function in infectious keratitis.

Corneal HOA has been evaluated and analysed using AS-OCT for various corneal and ocular surface diseases^4–10,14,15^, and the HOA value is considered an important quantitative indicator of corneal irregular astigmatism, not only in keratoconus but also in various corneal opacities^4–9^. Conventional instruments use Meier images to measure irregular astigmatism of the anterior surface and have difficulty assessing corneal irregular astigmatism in eyes with high corneal opacity. Advances in swept-source AS-OCT instruments have enabled the evaluation of corneal irregular astigmatism in such opaque eyes, including both the anterior and posterior surfaces of the cornea, as it uses an infrared light source^16^. However, previous studies focused on evaluations during the scarring period or when the condition had settled^4–7,9^. In the present study, HOA was evaluated over time from the acute phase of infectious keratitis before treatment to the scar phase after healing. The corneal shape was traced, and HOA could be calculated using AS-OCT, even in eyes with high corneal opacities and morphological changes. The HOA values during the posttreatment scar phase were consistent with those in a previous report^4^, whereas the pretreatment HOA for infectious keratitis was very large. Infectious keratitis often causes symptoms, such as eye pain, tearing, and eye discharge, as well as significant vision loss. Extremely large pretreatment HOAs could be a new finding, as visual deterioration was quantitatively shown based not only on the subjective test of visual acuity, which was affected by factors other than corneal condition, but also on morphological changes with corneal tomographic analysis.

Corneal HOA was analysed based on the aetiology, and among FK, BK, and AK, pretreatment HOA was the largest in FK, although the difference was not significant. This trend was also observed after treatment; however, the posttreatment total and anterior corneal HOAs were significantly larger in FK than in AK, and the posterior corneal HOAs were significantly larger in FK than those in BK. This result is similar to that of a previous report^4^ and could indicate that FK can penetrate the deep layers of the cornea and significantly alter the corneal stroma^17,18^. Patients with AK also tended to have a smaller pretreatment posterior corneal HOA than those with BK, FK, and HSK. This may reflect the fact that *Acanthamoeba* first infects the corneal epithelium and then invades the superficial layer of the corneal stroma^19^. Although smear microscopic examination and culture are essential methods for diagnosing infectious keratitis^1,20^, scanning AS-OCT to evaluate HOA values and careful examination of cross-sectional images^20–24^ may assist in understanding the pathophysiology of infectious keratitis.

We further explored whether the factors affecting visual acuity after the treatment of infectious keratitis could be predicted from pretreatment factors such as HOA. Infectious keratitis often makes it difficult to predict the final corneal state after scar healing. However, if visual prognosis can be predicted based on the pretreatment status, it could be very useful for therapeutic planning and patient explanation. Multivariable analysis demonstrated that pretreatment HOA (anterior, 6 mm), pretreatment logMAR BSCVA, and age were associated with posttreatment visual acuity. Association with large anterior corneal HOA and poor pretreatment visual acuity suggest a more severe condition of infectious keratitis at the initial visit. It is plausible that the more severe the infectious keratitis is before treatment, the greater is the probability of visual impairment after treatment. Subjective visual acuity is greatly affected by various factors other than corneal structure, whereas HOA is an objective indicator that can represent corneal visibility based on corneal tomography and can be easily measured using AS-OCT. For example, intraocular pressure, retinal nerve fibre layer, and visual field are assessed and monitored in all patients with glaucoma as they are universal indicators that represent glaucoma. As in these indicators, corneal HOA reflects the severity of infectious keratitis and changes during treatment and correlates with visual acuity; therefore, corneal HOA can be used as an objective biomarker that can represent the visual function in both mild and severe cases. Furthermore, the HOA values and topography map patterns can be useful in explaining the condition and alteration of infectious keratitis in patients, as well as in improving adherence to topical antimicrobial agents.

Regarding age, the older patients enrolled in this study tended to have poorer pretreatment visual acuity and larger HOAs. In general, these factors tend to worsen in older patients^25^. Therefore, the possibility of bias exists, and further studies on the effect of age may be needed. Moreover, we examined the topographic map patterns and found that there were cases in which the topographic map pattern changed between the acute phase of infection and the posttreatment scar state. In the acute phase, the most frequent topographic map pattern was an asymmetric pattern, followed by posterior irregular and protrusion patterns due to inflammatory cell infiltration and corneal edema. In the posttreatment scarring phase, the minimal change and asymmetric patterns were more common, and the proportion of posterior irregular and protrusion patterns decreased. The topographic map pattern affected the corneal HOA and posttreatment visual acuity; patients with asymmetric and protrusion patterns had larger HOA and poorer posttreatment visual acuity. Patients with a minimal change pattern had a smaller HOA, and this pattern had a smaller effect on visual acuity. These results indicate that a minimal change topographic map pattern and a small corneal HOA after treatment are predictive of better posttreatment visual acuity. However, since the topographic map patterns can change before and after treatment, visual acuity prognosis cannot be determined based on the pretreatment topographic map patterns alone. Multivariable analysis in the current study showed that a large pretreatment HOA is indicative of poor posttreatment visual prognosis. This supports the finding that corneal HOA evaluation would be more useful than topographic map patterns in inferring visual prognosis after infectious keratitis.

Our study had some limitations. First, corneal HOAs are not a common index in corneal practice, as AS-OCT is used only in limited hospitals. To solve this problem, we recently developed analysis software that shows corneal HOAs, topographic maps, densitometry data, and Landolt optotype simulation images. The software is useful for evaluating corneal HOA by medical staff and for showing results to patients and reinforcing adherence to medications. Second, the current study only showed that visual improvement was associated with corneal HOA reduction by treatment. It seems apparent, as it was obvious that the infectious keratitis had resolved. However, corneal HOAs represent the precise optical functional index of the cornea; thus, they can be an objective optical marker for evaluating the efficacy of conventional and novel treatments for minimizing corneal scarring. Third, infectious keratitis itself can influence the refractive index of the cornea by changing the hydration status of the cornea^26,27^, and the HOA values can also be affected. Theoretically, an increase in the refractive indices from 1.376 to 1.391 and 1.406 (estimated maximum change, based on previous reports^26–29^) will increase the corneal HOAs by 2.1% and 4.3%, respectively, for a 4.0 mm diameter^30^. The changes in the refractive indices appear to have a minimal influence on the calculation of corneal HOAs. We will need to adjust for corneal edema, although this study showed a significant correlation between logMAR visual acuity and corneal HOAs both before and after treatment. Fourth, this was a retrospective study. Thus, cases in which the AS-OCT images were unavailable at all set time points were excluded. This may have potentially induced selection bias.

In conclusion, corneal HOAs increase in the acute phase of infectious keratitis and decrease with the resolution of infection following antimicrobial treatment. The decrease in HOA is significantly correlated with visual improvement, indicating that corneal HOA can be used as an objective marker to represent the visual function in infectious keratitis. Furthermore, pretreatment anterior corneal HOA is associated with visual outcomes. Thus, the use of corneal HOA in treating infectious keratitis can potentially help improve visual prognosis by providing an objective index and facilitating precision medicine in corneal clinics.

## Supplementary Information

Supplementary Tables.

## Data Availability

The datasets generated during and/or analysed during the current study are available from the corresponding author on reasonable request.
