# Supplementary material for: Changes in corneal higher-order aberrations during treatment for infectious keratitis
Source: Sci Rep. 2023 Jan 16;13:848. doi: 10.1038/s41598-023-28145-7 (PMC9842715; doi:10.1038/s41598-023-28145-7)
Supplement: Supplementary file 1 — Supplementary Tables. [file 41598_2023_28145_MOESM1_ESM.pdf]

Supplementary Information

Changes in Corneal Higher-Order Aberrations During Treatment for Infectious Keratitis

Takehiro Matsumura[1,2,*], Takefumi Yamaguchi[1], Takanori Suzuki[1], Yurina Ogiwara[1], Yoshihiro Takamura[2], Masaru Inatani[2], and Jun Shimazaki[1,3]

[1]Department of Ophthalmology, Tokyo Dental College Ichikawa General Hospital, Chiba, Japan

[2]Department of Ophthalmology, Faculty of Medical Sciences, University of Fukui, Fukui, Japan

[3]Cornea Center Eye Bank, Tokyo Dental College Ichikawa General Hospital, Chiba, Japan

[*]Correspondence and requests for materials should be addressed to:

Takehiro Matsumura

Department of Ophthalmology, Tokyo Dental College Ichikawa General Hospital

5-11-13 Sugano, Ichikawa, Chiba 272-8513, Japan

Phone: +81-47-322-0151

Fax: +81-47-322-6786

E-mail: takebou_mail@yahoo.co.jp

Supplementary Table S1. Corneal higher-order aberrations before and after treatment for each grade of corneal opacity

	Corneal opacity grade				P value					
**Before treatment**	**Grade 1** (n = 8)	**Grade 2** (n = 23)	**Grade 3** (n = 67)		***P*[a]**	***P*[b]**	***P*[c]**			
HOA (4 mm)										
Total	0.91 (0.60–1.30)	1.50 (1.20–2.27)	2.81 (1.29–4.92)		0.074	0.0089	0.074			
Anterior	0.65 (0.57–1.05)	1.20 (0.88–2.39)	2.76 (1.27–4.59)		0.059	0.0048	0.0368			
Posterior	0.14 (0.10–0.61)	0.54 (0.33–0.75)	0.65 (0.43–1.11)		0.24	0.037	0.28			
HOA (6 mm)										
Total	1.63 (1.17–2.32)	2.62 (1.56–5.14)	4.20 (2.38–7.92)		0.11	0.0028	0.079			
Anterior	1.28 (1.07–2.45)	2.22 (1.66–5.26)	4.31 (2.37–8.97)		0.24	0.0040	0.035			
Posterior	0.28 (0.20–0.98)	0.90 (0.63–1.26)	0.95 (0.70–1.73)		0.083	0.0099	0.57			
**After treatment**	**Grade 0** (n = 16)	**Grade 1** (n = 42)	**Grade 2** (n = 27)	**Grade 3** (n = 13)	***P*[d]**	***P*[e]**	***P*[f]**	***P*[a]**	***P*[b]**	***P*[c]**
HOA (4 mm)										
Total	0.40 (0.31–0.93)	1.15 (0.46–1.68)	1.30 (0.78–2.94)	2.36 (1.25–3.65)	0.039	0.0034	0.0007	0.34	0.021	0.42
Anterior	0.42 (0.30–1.02)	1.19 (0.47–1.77)	1.40 (0.79–2.67)	2.40 (1.02–3.57)	0.042	0.0040	0.0013	0.40	0.036	0.60
Posterior	0.09 (0.06–0.26)	0.13 (0.08–0.27)	0.23 (0.10–0.46)	0.66 (0.34–0.92)	0.67	0.054	0.0004	0.19	0.0003	0.027
HOA (6 mm)										
Total	0.82 (0.59–1.58)	2.02 (0.97–4.18)	3.23 (2.00–5.46)	4.26 (2.33–7.14)	0.0088	< 0.0001	0.0004	0.087	0.043	0.77
Anterior	0.85 (0.60–1.64)	2.05 (0.99–4.08)	3.04 (1.75–5.65)	4.56 (2.25–7.43)	0.012	0.0001	0.0006	0.13	0.064	0.66
Posterior	0.20 (0.17–0.30)	0.23 (0.17–0.39)	0.42 (0.21–0.91)	0.99 (0.48–1.50)	0.89	0.043	0.0003	0.097	0.0002	0.071

HOA = higher-order aberration.

The values are presented as median (interquartile range) (μm).

[a]*P* values were compared using the Steel–Dwass test for non-parametric multiple comparison between grade 1 and grade 2; [b]*P* values were between grade 1 and grade 3; [c]*P* values were between grade 2 and grade 3; [d]*P* values were between grade 0 and grade 1; [e]*P* values were between grade 0 and grade 2; and [f]*P* values were between grade 0 and grade 3.

Supplementary Table S2. Correlation between corneal higher-order aberration and corneal opacity area

	Corneal infiltration area (before treatment)		Corneal scar area (after treatment)	
	**ρ**	***P* value**	**ρ**	***P* value**
HOA (4 mm)				
Total	0.416	< 0.0001	0.467	< 0.0001
Anterior	0.439	< 0.0001	0.458	< 0.0001
Posterior	0.396	< 0.0001	0.492	< 0.0001
HOA (6 mm)				
Total	0.404	< 0.0001	0.531	< 0.0001
Anterior	0.400	< 0.0001	0.520	< 0.0001
Posterior	0.402	< 0.0001	0.502	< 0.0001

HOA = higher-order aberration.
ρ = Spearman's rank correlation coefficient.

Supplementary Table S3. Corneal higher-order aberration based on the presence or absence of central corneal opacity

	Infiltrate at the centre of the cornea (before treatment)			Scar at the centre of the cornea (after treatment)		
	**No**	**Yes**	***P* value**	**No**	**Yes**	***P* value**
HOA (4 mm)						
Total	1.33 (0.60–2.10)	2.86 (1.53–5.51)	< 0.0001	0.71 (0.36–1.48)	1.34 (0.80–2.62)	0.0024
Anterior	1.10 (0.58–2.27)	2.86 (1.29–5.23)	< 0.0001	0.72 (0.37–1.54)	1.37 (0.87–2.43)	0.0017
Posterior	0.43 (0.13–0.70)	0.73 (0.52–1.12)	< 0.0001	0.10 (0.06–0.27)	0.29 (0.15–0.55)	< 0.0001
HOA (6 mm)						
Total	2.39 (1.24–3.29)	5.68 (2.78–9.00)	< 0.0001	1.62 (0.83–3.14)	2.71 (1.88–5.03)	0.0093
Anterior	2.37 (1.15–3.71)	5.50 (2.34–9.13)	< 0.0001	1.53 (0.87–2.96)	2.83 (1.77–5.27)	0.0081
Posterior	0.66 (0.34–1.10)	1.01 (0.83–1.76)	< 0.0001	0.20 (0.17–0.34)	0.43 (0.26–0.97)	< 0.0001

HOA = higher-order aberration.
The values are presented as median (interquartile range) (μm).
P values were compared between with or without central corneal lesions using the Mann–Whitney U test.
Central corneal opacity (infiltrate or scar) was defined as a lesion within 3 mm of centre of the cornea.

Supplementary Table S4. Correlations between the corneal higher-order aberration change and visual improvement after treatment

	**ρ**	***P* value**
ΔHOA (4 mm)		
Total	0.166	0.113
Anterior	0.147	0.163
Posterior	0.231	0.027
ΔHOA (6 mm)		
Total	0.228	0.029
Anterior	0.217	0.038
Posterior	0.252	0.015

HOA = higher-order aberration.
ρ = Spearman's rank correlation coefficient.
One patient each with proliferative diabetic retinopathy, advanced glaucoma, and moderate-to-severe cataract were excluded from the analysis of visual acuity.
